# Supplementary material for: Biomarkers Associated with Lymph Nodal Metastasis in Endometrioid Endometrial Carcinoma
Source: Cancers (Basel). 2022 Apr 27;14(9):2188. doi: 10.3390/cancers14092188 (PMC9099548; doi:10.3390/cancers14092188)
Supplement: Supplementary file 1 [file cancers-14-02188-s001.zip › cancers-1663821-supplementary.pdf]

Supplementary data

Table S1. Detailed histological and molecular characteristics of patients with EEC treated at Institut Bergonie selected after matching and quality control (N=28), France, 2010–2017.

ID	N status	Grade	Myometrial invasion	LVSI	Stromal reaction	MELF pattern	Inflammatory inflammation	Peri nervous invasion	*POLE* pathogenic mutation	p53 IHC	*TP53* mutation	*TP53* mutation's pathogenicity	MMR status (IHC)	Molecular classification group	CTNNB1 mutation	CTNNB1 mutation's pathogenicity
1	N+	2	> 50%	Substantial	Presence	Absence	Absence	Presence	Absence	wild type	exon3:c.98C>G	Non-pathogenic	Proficient	NSMP		
2	N+	2	> 50%	Substantial	Presence	Presence	Absence	Absence	Absence	wild type			Proficient	NSMP		
3	N+	3	> 50%	Substantial	Absence	Absence	Presence	Absence	Absence				Deficient	Hypermutated (MSI)		
4	N+	2	> 50%	Absent	Absence	Absence	Absence	Absence	Absence				Deficient	Hypermutated (MSI)		
5	N+	1	≤ 50%	Substantial	Presence	Presence	Absence	Presence	Absence	wild type	exon5:c.522A>G; exon3:c.98C>G	Non-pathogenic	Deficient	Hypermutated (MSI)		
6	N+	3	> 50%	Substantial	Presence	Presence	Absence	Absence	Absence	wild type	exon3:c.98C>G	Non-pathogenic	Deficient	Hypermutated (MSI)		
7	N+	2	≤ 50%	Absent	Absence	Absence	Absence	Absence	Absence	wild type	exon5:c.522A>G exon3:c.98C>G	Non-pathogenic	Proficient	NSMP	exon3:c.98C>A exon3:c.98C>T	Pathogenic
8	N+	2	> 50%	Substantial	Presence	Presence	Presence	Absence	Absence	wild type			Deficient	Hypermutated (MSI)		
10	N+	2	≤ 50%	Non-substantial	Presence	Presence	Absence	Absence	Absence	wild type	exon3:c.98C>G	Non-pathogenic	Proficient	NSMP		
11	N+	2	> 50%	Non-substantial	Absence	Absence	Absence	Absence	Absence	wild type			Deficient	Hypermutated (MSI)		
12	N+	3	> 50%	Non-substantial	Presence	Presence	Presence	Presence	Absence	Abnormal	exon6:c.655G>A	Pathogenic	Proficient	Serous like (TP53 mutation)		
13	N+	3	> 50%	Substantial	Presence	Absence	Absence	Presence	Absence	wild type	exon3:c.98C>G	Non-pathogenic	Deficient	Hypermutated (MSI)		
14	N+	2	≤ 50%	Substantial	Presence	Presence	Presence	Absence	Absence	wild type			Deficient	Hypermutated (MSI)		
15	N+	2	> 50%	Substantial	Presence	Presence	Absence	Absence	Absence	wild type	exon7:c.700C>T	Pathogenic	Proficient	Serous like (TP53 mutation)	exon3:c.110C>T	Pathogenic
16	N+	3	≤ 50%	Non-substantial	Presence	Presence	Presence	Absence	Absence	wild type	exon3:c.98C>G	Non-pathogenic	Deficient	Hypermutated (MSI)		
17	N+	2	≤ 50%	Absent	Presence	Presence	Presence	Absence	Absence	Abnormal	exon6:c.655G>A	Pathogenic	Proficient	Serous like (TP53 mutation)		

ID	N status	Grade	Myometrial invasion	LVSI	Stromal reaction	MELF pattern	Inflammatory inflammation	Peri nervous invasion	*POLE* pathogenic mutation	p53 IHC	*TP53* mutation	*TP53* mutation's pathogenicity	MMR status (IHC)	Molecular classification group	CTNNB1 mutation	CTNNB1 mutation's pathogenicity
32	N-	3	≤ 50%	Absent	Absence	Absence	Absence	Absence	Absence	wild type			Proficient	NSMP		
33	N-	2	≤ 50%	Non-substantial	Presence	Presence	Presence	Absence	Absence	wild type	exon3:c.98C>G	Non-pathogenic	Proficient	NSMP		
39	N-	2	> 50%	Absent	Presence	Absence	Absence	Absence	Absence	wild type			Proficient	NSMP	exon3:c.110C>T	Pathogenic
40	N-	2	> 50%	Absent	Absence	Absence	Absence	Absence	Absence	wild type	exon5:c.522A>G exon3:c.98C>G	Non-pathogenic	Deficient	Hypermutated (MSI)		
46	N-	2	> 50%	Absent	Absence	Absence	Presence	Absence	Absence	wild type			Deficient	Hypermutated (MSI)		
49	N-	3	> 50%	Substantial	Presence	Presence	Presence	Absence	Absence	wild type			Deficient	Hypermutated (MSI)		
50	N-	2	> 50%	Absent	Absence	Absence	Absence	Absence	Absence	wild type			Proficient	NSMP	exon3:c.134C>T	Pathogenic
51	N-	2	> 50%	Absent	Presence	Presence	Presence	Presence	Absence	wild type	exon5:c.522A>G	Non-pathogenic	Proficient	NSMP	exon3:c.98C>Aexon3:c.98C>T	Pathogenic
53	N-	1	≤ 50%	Absent	Absence	Absence	Absence	Absence	Absence	wild type			Proficient	NSMP	exon3:c.98C>A exon3:c.98C>T	Pathogenic
56	N-	3	> 50%	Substantial	Presence	Absence	Presence	Absence	Absence	wild type			Deficient	Hypermutated (MSI)		
59	N-	2	> 50%	Absent	Absence	Absence	Absence	Absence	Absence	wild type	exon3:c.98C>G	Non-pathogenic	Proficient	NSMP	exon3:c.110C>T	Pathogenic

Table S2. Comparison of histopathological characteristics and molecular patterns of cluster A (*n* =8), cluster B (*n* =10) and cluster C (*n* =11) defined in unsupervised analysis, Institut Bergonie, 2010–2017.

**Pathological characteristics**	**Cluster A (*n* =7)**		**Cluster B (*n* =10)**		**Cluster C (*n* =11)**		
	n (%)	Mean (SD)	n (%)	Mean (SD)	n (%)	Mean (SD)	***p*-value**[1]
**Lymph node involvement**							0.007
**N0**	0 (0.0)		4 (40.0)		8 (72.7)		
**N1**	7 (100.0)		6 (60.0)		3 (27.3)		
**Histological grade**							0.14
Low grade (grade 1 & grade 2)	2 (28.6)		5 (50,0)		1 (9.1)		
High grade (grade 3)	5 (71.4)		5 (50,0)		10 (90.9)		
**Myometrial invasion**							0.9
<= 50%	3 (42.9)		3 (30.0)		4 (36.4)		
> 50%	4 (57.1)		7 (70.0)		7 (63.6)		
**Angioinvasion**							0.05
Presence	5 (74.4)		8 (80.0)		3 (27.3)		
Absence	2 (28.6)		2 (20.0)		8 (72.7)		
**Number of angioinvasion (if presence of angioivasion)**		4.4 (4.0)		11.7 (12.3)		1.4 (2.8)	
**Stromal Reaction**							0.12
Presence	5 (71.4)		8 (80.0)		4 (36.4)		
Absence	2 (28.6)		2 (20.0)		7 (63.6)		
**MELF pattern**							0.3
Presence	4 (57.1)		6 (60.0)		3 (27.3)		
Absence	3 (42.9)		4 (40.0)		8 (72.7)		
**Inflammatory infiltration**							0.6
Presence	3 (42.9)		5 (50.0)		3 (27.3)		
Absence	4 (57.1)		5 (50.0)		8 (72.7)		
**Peri nervous invasion**							0.8
Presence	2 (28.6)		1 (10.0)		2 (18.2)		
Absence	5 (71.4)		9 (90.0)		9 (81.8)		
**Molecular classification (TCGA)**							
Ultramutated							
Hypermutated							
Serous like							
Non specific molecular profile							

Table S3 Comparison of histopathological characteristics and molecular patterns of EEC from cluster C without lymph node involvement (*n* =8) and with lymph node involvement (*n* =3), Institut Bergonie, 2010–2017.

Pathological characteristics	EEC with negative lymph nodes (*n* =8)		EEC with positive lymph nodes (*n* =3)	
	n (%)	Mean (SD)	n (%)	Mean (SD)
**Histological grade**				
Low grade (grade 1 & grade 2)	7 (87.5)		3 (100.0)	
High grade (grade 3)	1 (12.5)		0 (0.0)	
**Myometrial invasion**				
≤ 50%	2 (25.0)		2 (66.7)	
> 50%	6 (75.0)		1 (33.3)	
**Angioinvasion**				
Absence	8 (100.0)		0 (0.0)	
Non-substantial	0 (0.0)		1 (33.3)	
Substantial	0 (0.0)		2 (66.7)	
**Stromal Reaction**				
Presence	2 (25.0)		2 (66.7)	
Absence	6 (75.0)		1 (33.3)	
**MELF pattern**				
Presence	7 (87.5)		1 (33.3)	
Absence	1 (12.5)		2 (66.7)	
**Inflammatory infiltration**				
Presence	2 (25.0)		1 (33.3)	
Absence	6 (75.0)		2 (66.7)	
**Peri nervous invasion**				
Presence	1 (12.5)		1 (33.3)	
Absence	7 (87.5)		2 (66.7)	
**Molecular classification group (TCGA)**				
Ultramutated (*POLE* mutation)	0 (0.0)		0 (0.0)	
Hypermutated (MSI)	2 (25.0)		3 (100.0)	
Serous like (*TP53* mutation)	0 (0.0)		0 (0.0)	
Non specific molecular profile	6 (75.0)		0 (0.0)	

Table S4. Fifty-four genes signature to discriminate -N+ and N- patients, Institut Bergonie, 2010–2017.

SYMBOL	DESCRIPTION
ABCC9	ATP-binding cassette, sub-family C (CFTR/MRP), member 9
ACTA2	actin, alpha 2, smooth muscle, aorta
ACTG2	actin, gamma 2, smooth muscle, enteric
ALDH1A2	aldehyde dehydrogenase 1 family, member A2
ARSI	arylsulfatase family, member I
ASPN	asporin
BCHE	butyrylcholinesterase
BNC2	basonuclin 2
C9ORF171	chromosome 9 open reading frame 171
CACNB2	calcium channel, voltage-dependent, beta 2 subunit
CCDC65	coiled-coil domain containing 65
CHRDL2	chordin-like 2
CNN1	calponin 1, basic, smooth muscle
DDR2	discoidin domain receptor tyrosine kinase 2
DES	desmin
DPP6	dipeptidyl-peptidase 6
EFEMP1	EGF containing fibulin-like extracellular matrix protein 1
EXTL1	exostosin-like glycosyltransferase 1
FLNC	filamin C, gamma
FNDC5	fibronectin type III domain containing 5
GAS2L2	growth arrest-specific 2 like 2
GUCY2F	guanylate cyclase 2F, retinal
HABP2	hyaluronan binding protein 2
HSPB6	heat shock protein, alpha-crystallin-related, B6
JPH2	junctophilin 2
KCNAB1	potassium voltage-gated channel, shaker-related subfamily, beta member 1
KIF5A	kinesin family member 5A
LMOD1	leiomodin 1 (smooth muscle)
MEP1A	meprin A, alpha (PABA peptide hydrolase)
MGP	matrix Gla protein
MRGPRF	MAS-related GPR, member F
MUM1L1	melanoma associated antigen (mutated) 1-like 1
MYH11	myosin, heavy chain 11, smooth muscle
MYOCD	myocardinmyocardin
NAALAD2	N-acetylated alpha-linked acidic dipeptidase 2
PAGE4	P antigen family, member 4 (prostate associated)
PDLIM3	PDZ and LIM domain 3
PRSS46	protease, serine, 46
PRSS50	protease, serine, 50
PTGFR	prostaglandin F receptor (FP)
RERG	RAS-like, estrogen-regulated, growth inhibitor
ROPN1L	rhophilin associated tail protein 1-like
RSPO1	R-spondin 1
RSPO3	R-spondin 3

SGCA	sarcoglycan, alpha (50kDa dystrophin-associated glycoprotein)
SLC22A3	solute carrier family 22 (organic cation transporter), member 3
SPARCL1	SPARC-like 1 (hevin)
SSC5D	scavenger receptor cysteine rich domain containing (5 domains)
TAGLN	transgelin
TCEAL5	transcription elongation factor A (SII)-like 5
TNXB	tenascin XB
TRIM55	tripartite motif containing 55
UNC5C	unc-5 homolog C (C. elegans)
WT1	Wilms tumor 1

Table S5. Confusion matrix of cross validated 54 genes.

		EXPECTED		
		N+	*N-*	*TOT*
OBSERVED	*N+*	6	1	7
	N-	1	7	8
	TOT	7	8	15

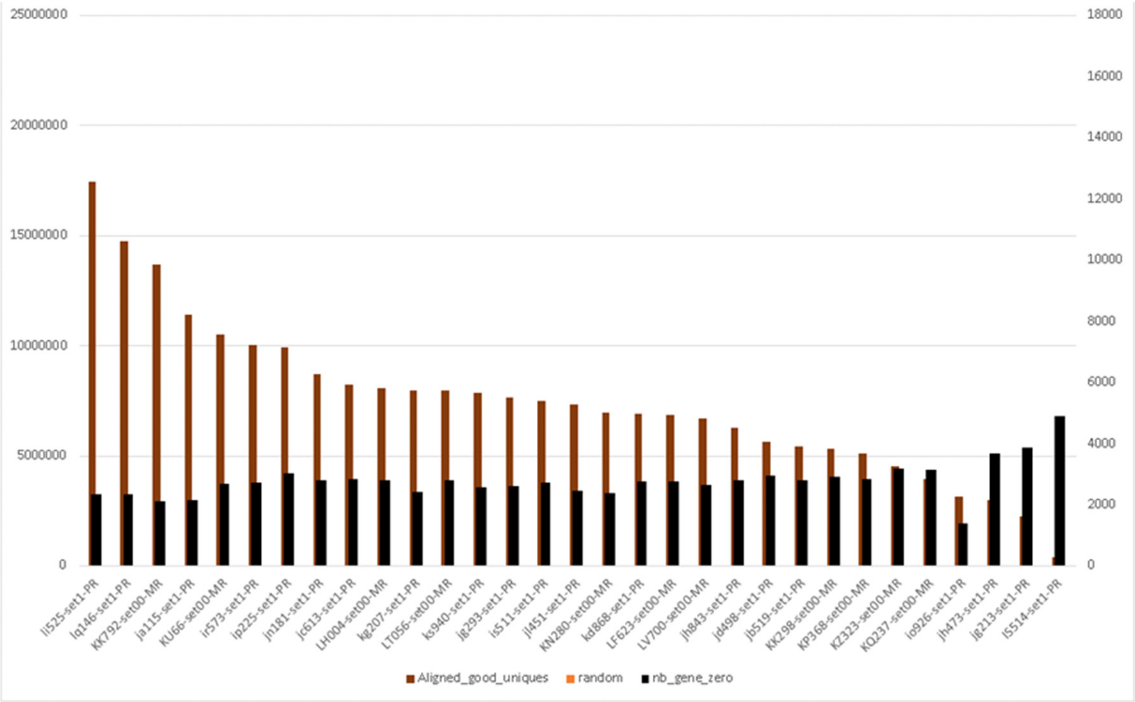

Figure S1. Quality control proportion on gene to zero according to coverage.

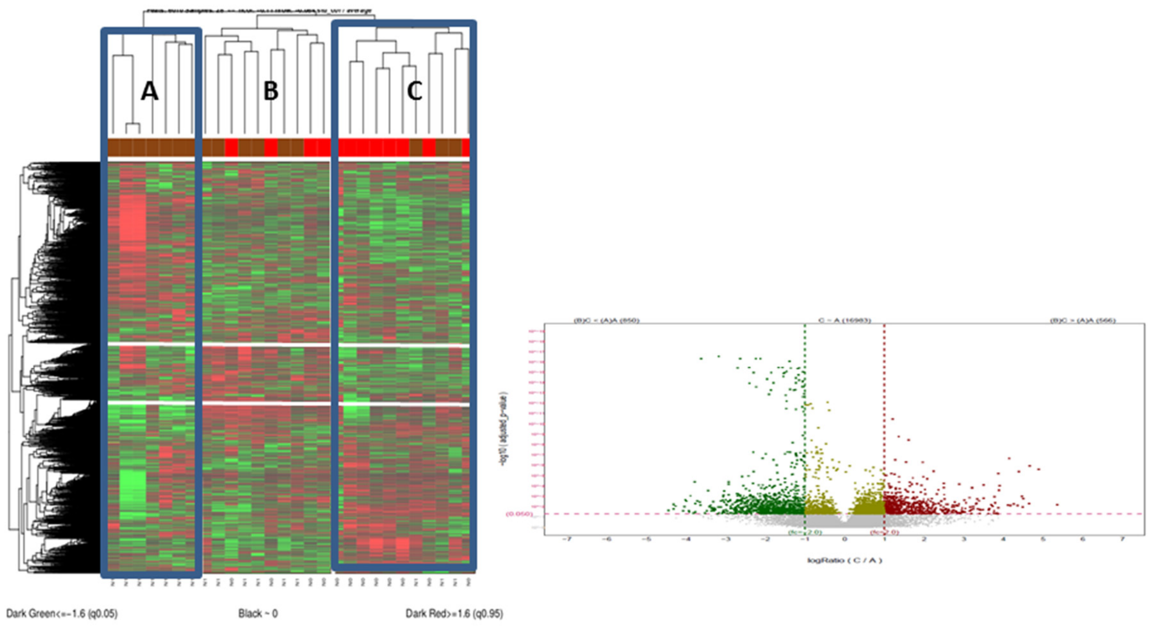

Figure S2. Differential gene expression on cluster A and C found 1416 genes, Institut Bergonie, 2010–2017.

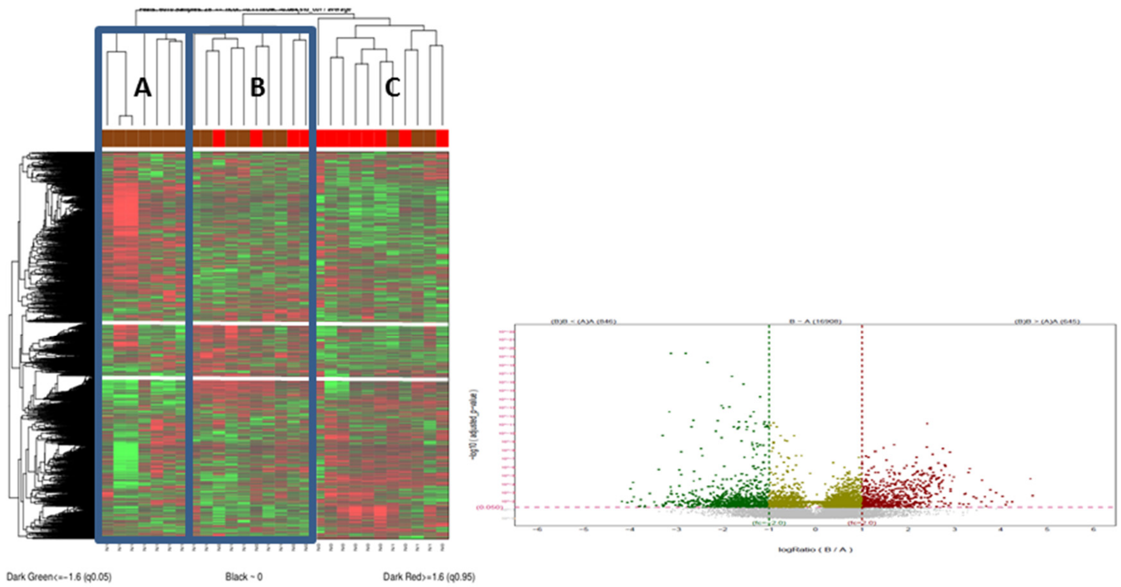

Figure S3. Differential gene expression of cluster A and B found 1491 genes, Institut Bergonie, 2010–2017.

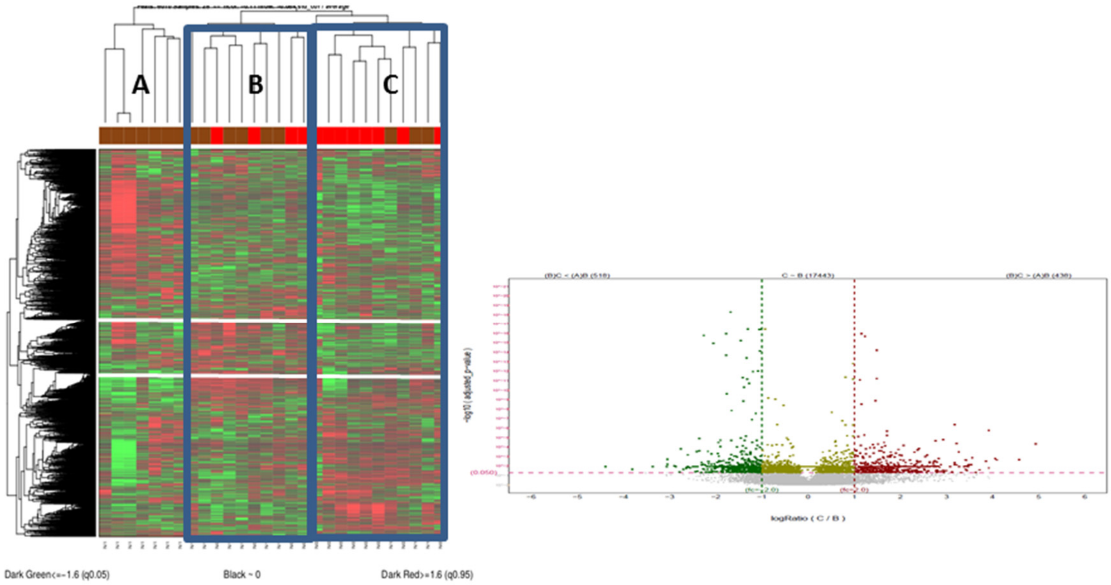

Figure S4. Differential gene expression of cluster B and C found 956 genes, Institut Bergonie, 2010–2017.

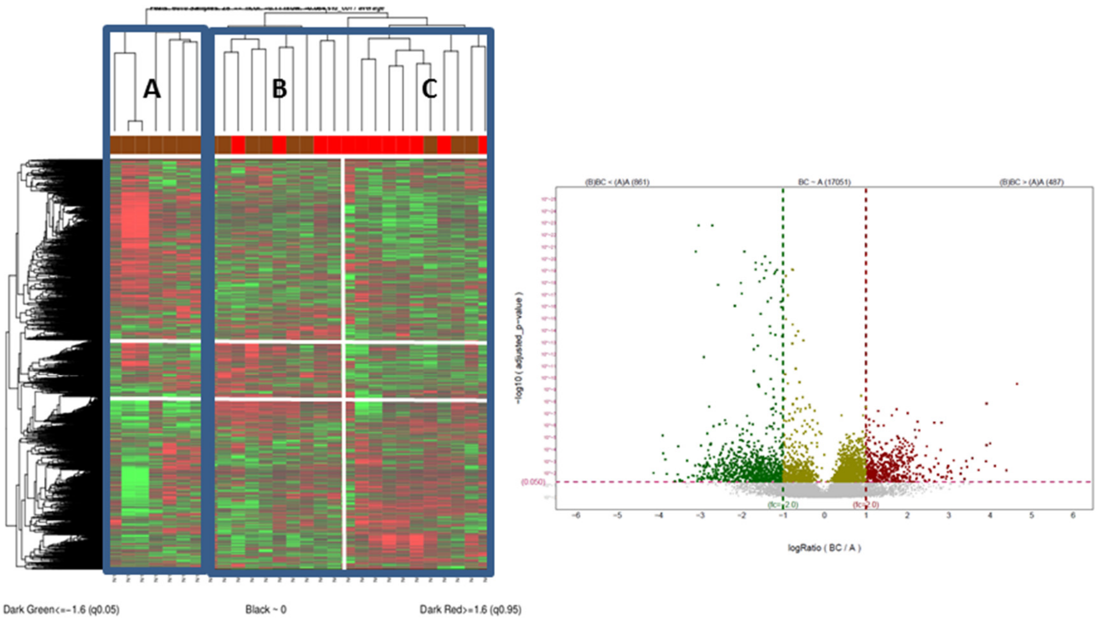

Figure S5. Differential gene expression of cluster A and B+C found 1348 genes, Institut Bergonie, 2010–2017.

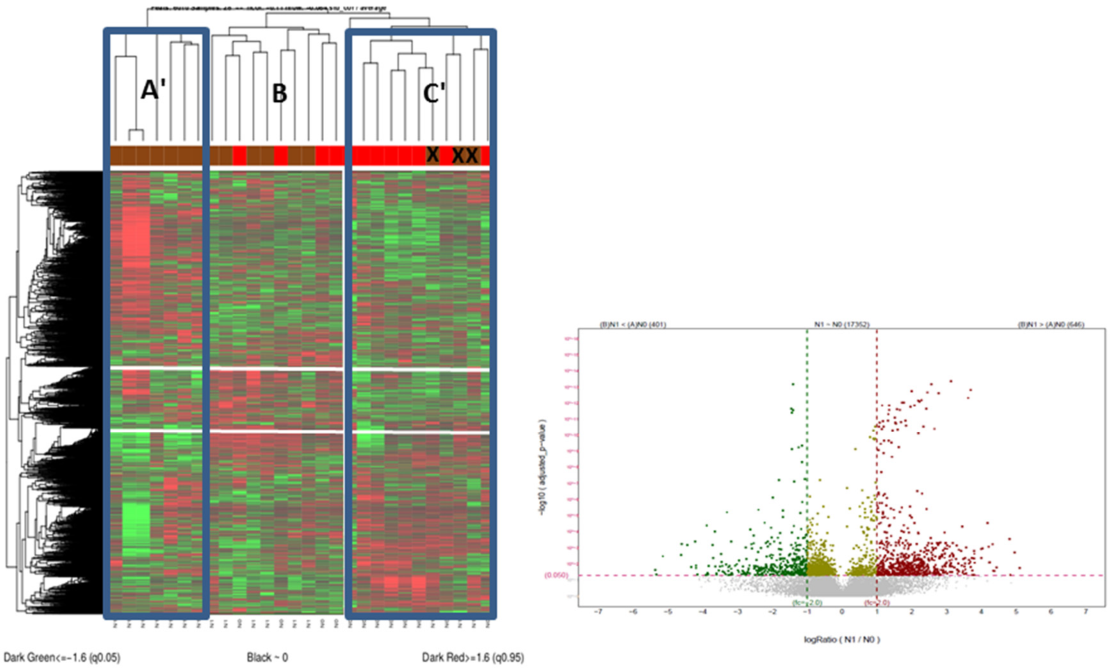

Figure S6. Differential gene expression of cluster A and C (N-patients only in C group) found 1047 genes, Institut Bergonie, 2010–2017.
